# SLEEP DURATION MEDIATES THE RELATIONSHIP BETWEEN LETTER FLUENCY AND COGNITION

**DOI:** 10.1093/geroni/igac059.2082

**Published:** 2022-12-20

**Authors:** Hyeon Jung Kim, Subhranshu Banerjee, Julie Blaskewicz Boron

**Affiliations:** University of Nebraska Omaha, Omaha, Nebraska, United States; University of Nebraska Omaha, Omaha, Nebraska, United States; University of Nebraska, Omaha, Omaha, Nebraska, United States

## Abstract

Changes in cognition are a normative part of aging. Verbal fluency is widely used for assessing cognitive function, including predicting mild cognitive impairment. As people age, sleep duration and quality decrease. This has been associated with cognitive decline; individuals with low-quality sleep (fewer hours and difficulties with initiation or maintenance of sleep) are more likely to complain of poorer cognition. Lack of sleep is detrimental to verbal fluency (word production). Longer sleep has been linked to memory deficits and is a potential indicator of mild cognitive impairment. This study aimed to address the relationship between verbal fluency and cognitive function as mediated by sleep duration. Participants included 31 middle-aged adults (female, n=19;age=53.55±7.18yrs) and 24 older adults (female=14; age=70.33±3.69yrs). The MoCA assessed cognitive functioning. Verbal fluency (90 seconds each) was measured via category word (animals; semantic verbal fluency) and letter word (phonemic verbal fluency; F,A,S). Mediation models used Baron and Kenny’s (1986) approach for estimating mediation effects, controlling for age and education. Category results were non-significant. However, sleep duration partially mediated the relationship between letter fluency for each letter and cognitive function, respectively (F:F(2,52)=7.31, p=.002, R2=.22; A: F(2,52)=3.80, p=.029, R2=.18; S: F(2,52)=5.47, p=.007, R2=.17). Sleep hours were negatively associated with letter fluency, meaning fewer hours of sleep improved fluency performance, which in turn was positively associated with MOCA scores (F: b=-.378, 95%CI [-0.64, 0.02]; A: b=-.172, 95%CI [-0.42, 0.01]; S: b=-.24, 95%CI [-0.64, 0.02]. This coincides with the abovementioned literature and helps further understand how sleep and cognitive performance are related.

